# IL-6–Dependent STAT3 Activation and Induction of Proinflammatory Cytokines in Primary Sclerosing Cholangitis

**DOI:** 10.14309/ctg.0000000000000603

**Published:** 2023-06-01

**Authors:** Leona Dold, Leonie Frank, Philipp Lutz, Dominik J. Kaczmarek, Benjamin Krämer, Jacob Nattermann, Tobias J. Weismüller, Vittorio Branchi, Marieta Toma, Maria Gonzalez-Carmona, Christian P. Strassburg, Ulrich Spengler, Bettina Langhans

**Affiliations:** 1 Department of Internal Medicine I, University Hospital of Bonn, Bonn, Germany;; 2 German Center for Infection Research (DZIF), Partner Site Cologne-Bonn, Bonn, Germany;; 3 Department of Internal Medicine - Gastroenterology and Oncology, Vivantes Humboldt Hospital, Berlin, Germany;; 4 Department of General, Abdominal, Thoracic and Vascular Surgery, University Hospital Bonn, Bonn, Germany;; 5 Institute of Pathology, University Hospital Bonn, Bonn, Germany.

**Keywords:** Primary sclerosing cholangitis, proinflammatory CD4^+^ T cells, IL-6/STAT3 pathway, IFN gamma, IL-17A

## Abstract

**INTRODUCTION:**

Primary sclerosing cholangitis (PSC) is a rare cholestatic liver disease with periductal inflammation and fibrosis. Genetic studies suggest inflammatory cytokines and IL-6–dependent activation of transcription factor STAT3 as pivotal steps in PSC pathogenesis. However, details of inflammatory regulation remain unclear.

**METHODS:**

We recruited 50 patients with PSC (36 with inflammatory bowel disease, 14 without inflammatory bowel disease), 12 patients with autoimmune hepatitis, and 36 healthy controls to measure cytokines in the serum, bile, and immune cell supernatant using bead-based immunoassays and flow cytometry and immunohistochemistry to analyze phosphorylation of STATs in immune cells. Finally, we analyzed cytokines and STAT3 phosphorylation of T cells in the presence of JAK1/2 inhibitors.

**RESULTS:**

In PSC, IL-6 specifically triggered phosphorylation of STAT3 in CD4^+^ T cells and lead to enhanced production of interferon (IFN) gamma and interleukin (IL)-17A. Phospho-STAT3–positive CD4^+^ T cells correlated with systemic inflammation (C-reactive protein serum levels). Combination of immunohistology and flow cytometry indicated that phospho-STAT3–positive cells were enriched in the peribiliary liver stroma and represented CD4^+^ T cells with prominent production of IFN gamma and IL-17A. JAK1/2 inhibitors blocked STAT3 phosphorylation and production of IFN gamma and IL-6, whereas IL-17A was apparently resistant to this inhibition.

**DISCUSSION:**

Our results demonstrate systemic and local activation of the IL-6/STAT3 pathway in PSC. Resistance of IL-17A to STAT3-targeted inhibition points to a more complex immune dysregulation beyond STAT3 activation.

## Graphical abstract

**Figure:**
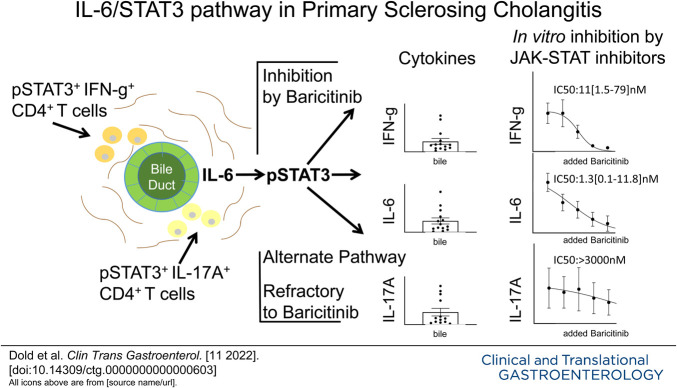

## INTRODUCTION

Primary sclerosing cholangitis (PSC) is a rare cholestatic liver disease characterized by inflammation and scarring around intrahepatic and extrahepatic bile ducts (1,2). PSC can progress to cholangiocellular carcinoma (CCA) (3) and is frequently associated with inflammatory bowel disease (IBD) and occasionally autoimmune hepatitis (AIH) (4). Currently, neither curative therapy nor treatment to slow PSC progression exists (5).

Although the exact pathogenesis remains unclear, proinflammatory T helper cells type 1 (TH1) (6,7) and type 17 (TH17) seem to play an essential role in PSC (8,9). Thus, the production of proinflammatory cytokines is considered to be pivotal for hepatic inflammation in PSC (10,11). Moreover, a recent gene-disease association study identified several genes comprising STAT3 (Signal Transducers and Activators of Transcription 3) and IL-6 as putative inflammatory hallmarks in human cholangiopathies (12). The idea of janus kinase (JAK)–STAT pathway activation in PSC is further supported by recent functional studies suggesting IL-6–dependent activation of STAT3 and subsequent release of inflammatory cytokines such as interferon (IFN) gamma and interleukin (IL)-17A as part of the pathogenesis (13,14).

The JAK-STAT pathway regulates cellular responses to cytokines, IFNs, and growth factors (15,16). It involves 4 janus kinases, JAK1-3 and TYK2, which phosphorylate STAT1-6 proteins. On phosphorylation, STAT proteins form homodimers and heterodimers and translocate to the nucleus, where they activate genes harboring their consensus sequence. STAT1 activates IFN gamma–producing TH1 cells (17,18), whereas IL-6 promotes TH17 differentiation through STAT3 activation in naïve CD4^+^ T cells (19). Both IL-6 and STAT3 enhance the expression and/or activation of IL-6, IL-17, and STAT3 through a positive feedback loop (20,21). Dysregulated IL-6-signaling contributes to the onset and persistence of several autoimmune diseases including IBD (22,23) and promotes the development of various cancer types, e.g., CCA (24). IL-6 also activates STAT1 and leads to the formation of STAT1/STAT3 heterodimers (15). Such mutual interactions act as IL-6 amplifier and again involve JAK-STAT pathways (25). However, details concerning the activation of JAK-STAT pathways in PSC must still be clarified.

In this study, we analyzed cytokines in the serum, bile, and immune cell supernatants from patients with PSC and compared the results with the STAT1/STAT3 activation state of immune cells in the blood and liver tissue. Of importance, we also performed *in vitro* blocking experiments of cytokines and STAT phosphorylation using broadly active JAK1/2 inhibitors.

## METHODS

### Patients and controls

In this study, we included 50 patients with early-stage PSC profiles. All patients were recruited from the Department of Internal Medicine I at the University Hospital of Bonn. The diagnosis of PSC was based on EASL clinical practice guidelines for cholestatic liver diseases (2). All patients with PSC had macroscopic disease, and none had features of a PSC/AIH variant. Concomitant IBD was diagnosed based on clinical criteria, and IBD phenotypes were classified (1,26) as ulcerative colitis, Crohn's disease (CD), or indeterminate colitis, in keeping with consensus guidelines (27,28). Forty-six of the 50 patients with PSC were on treatment with ursodeoxycholic acid (UDCA; 15 ± 5 mg/kg body weight). Seven patients had liver cirrhosis. Thirty-six patients had concomitant chronic IBD. IBD inflammatory activity was minimal, so that oral mesalazine (1.0–4.5 g/d) was the only given treatment needed in 28 (78%) patients.

Thirty-six sex-matched and age-matched healthy controls and 12 patients with AIH without features of PSC served as controls. All patients with AIH had AIH type 1 with detectable autoantibodies (antinuclear antibodies [ANAs]: n = 10, smooth muscle antibodies [SMAs]: n = 7). Healthy volunteers were recruited through the Bonn University blood banking service. Clinical features and demographic data of our patient groups and controls are summarized in Table 1. The study protocol followed the ethical guidelines of the Helsinki Declaration and had been approved by the local ethics committees (reference number 003/2020). Written informed consent was obtained from all participants before inclusion in this study. Owing to a limited availability of study specimens, we could unfortunately not perform all assays in every individual.

**Table 1.** Characteristics of the study cohorts

	PSC	AIH	Healthy controls
Total no. of individuals	50	12	36
Age (yr)	41 (20–67)	52 (28–76)	41 (20–79)
Sex (male/female)	28/22	4/8	23/13
PSC subphenotypes			
No. of patients with PSC without colitis	14	n.a.	n.a.
No. of patients with PSC with colitis	31 UC; 4 CD; 1 IC	n.a.	n.a.
Clinical parameters			
Amsterdam-Oxford score^a^	1.00 (0.22–3.52)	n.a.	n.a.
Transient elastography (kPa)	6.9 (3.8–75.0)	8.0 (5.5–9.2)	n.d. [<7.2]
Steatosis CAP (dB/m)	197.0 (105.0–363.0)	222.5 (206.0–289.0)	n.d. [<235]
AST (U/L)	33.0 (8.0–359.0)	29.0 (12.0–263.0)	n.d. [<50]
ALT (U/L)	36.0 (7.0–771.0)	29.0 (14.0–390.0)	n.d. [< 50]
AP (U/L)	141.0 (48.0–508.0)	69.5 (29.0–421.0)	n.d. [40–130]
Bilirubin (mg/dL)	0.66 (0.22–5.40)	0.60 (0.26–12.7)	n.d. [<1.4]
CRP (mg/L)	2.06 (0.67–32.46)	n.d.	n.d. [0–3]
Leukocytes (G/L)	6.33 (2.83–12.46)	5.82 (3.42–9.71)	n.d. [3.9–10.2]
IgG (g/L)	13.0 (6.9–19.4)	12.7 (9.0–25.1)	n.d. [7.0–16.0]
Platelets (G/L)	263 (45–497)	242 (158–408)	n.d. [150–370]
Treatments (no. of patients)	43 UDCA 27 Melasazine	4 UDCA 9 Antimetabolites 8 Corticosteroids	n.a.

(): Values are given as medians and ranges. [ ]: Values indicate reference ranges.

AIH, autoimmune hepatitis; ALT, alanine aminotransferase; AP, alkaline phosphatase; AST, aspartate aminotransferase; CAP, controlled attenuation parameter; CD, Crohn's disease; CRP, C-reactive protein; CU, ulcerative colitis; IgG, immunoglobulin G; IC, indeterminate colitis; n.a., not applicable; n.d., not done; PSC, primary sclerosing cholangitis; UDCA, ursodeoxycholic acid.

a Amsterdam-Oxford score: https://sorted.co/psc-calculator/.

### Reagents

Recombinant human IL-6 and IFN gamma (both carrier-free, Biolegend, London) and anti-CD3 and anti-CD28 (both Thermo Fisher Scientific, Germany) were used for *in vitro* stimulation. JAK inhibitors baricitinib, upadacitinib, and fedratinib were purchased from MedChemExpress (NJ).

### Cell preparations from peripheral blood

Two ml of heparinized blood was analyzed after red blood cell lysis (RBC; 1x Lyse/Fix Buffer; Biolegend) directly within 3 hours of blood sampling for analysis of phospho-STATs in the whole blood. From the remaining blood, peripheral blood mononuclear cells (PBMCs) were isolated by Ficoll-Paque density gradient centrifugation (PAA Laboratories, Cölbe, Germany) and cryopreserved in liquid nitrogen until analysis.

### Measurement of cytokines in the serum, bile, and cell culture supernatants

Cytokine analysis was performed in serum samples centrifuged from separate tubes with clotted venous blood and stored at −80°C until use. In PSC, bile samples were aspirated after intubation of bile ducts during endoscopic retrograde cholangiogra (ERC) before contrast medium was applied. Bile samples were immediately stored at −80°C. To study the induction of cytokines in cell culture supernatants, PBMCs were thawed and stimulated *in vitro* with anti-CD3 (1 μg/mL) plus anti-CD28 (0.5 μg/mL). After 24 hours, supernatants were collected and stored until use.

Cytokine concentrations in the serum, bile, and supernatants were measured through flow cytometry using the LEGENDplex HU Essential Immune Response Panel (Biolegend). Samples were analyzed on a FACSCanto II (BD Biosciences, Heidelberg, Germany), and evaluation was performed with the LEGENDplex cloud-based software. In line with the observations of Kemp et al (29), approximately 10% of bile samples had to be excluded from the analysis owing to high biliary viscosity with bead aggregation.

### Analysis of phospho-STAT1, phospho-STAT3, and cytokines in T cells

STAT activation was analyzed using commercially available PE-labeled antibodies against phospho-STAT1 (clone A17016B.Rec) and phospho-STAT3 (clone 13A3-1; both Biolegend) according to established protocols (https://www.biolegend.com/en-us/bio-bits/phospho-staining-and-intracellular-flow-cytometry). In addition to analysis of the whole blood allowing detection of phosphorylation from *in vivo* signaling, phospho-STATs were studied in PBMCs without prior stimulation and after *in vitro* stimulation (15 minutes at 37°C, 5% CO_2_) with recombinant cytokines (50 ng/mL of IL-6 for phospho-STAT3, 50 ng/mL each of IFN gamma plus IL-6 each for phospho-STAT1).

In brief, cells were washed with Cell Staining Buffer, and dead/viable cells were discriminated by Zombie Aqua™ staining (BioLegend). After 10 minutes, cells were stained with anti-CD3 (PE-Cy7–labeled), anti-CD4 (allophycocyanin-Cy7-labeled), and anti-CD8 (fluorescein-5-isothiocyanate-labeled) (all BioLegend). After washing with Permeabilization Wash Buffer (1X), cells were resuspended in True Phos Perm Buffer (both Biolegend) and incubated overnight at −20°C in the freezer. This protocol enabled to also measure cytokines without the need to add Golgi transport inhibitors (see Supplementary Figure 1, Supplementary Digital Content, http://links.lww.com/CTG/A950). Next day, cells were thawed, washed, and stained with anti–phospho-STAT1 and anti-STAT3 in Cell Staining Buffer, respectively. In detailed experiments, cells were further costained intracellularly with allophycocyanin-labeled anti–IFN gamma and BV-421–labelled anti–IL-17A (all Biolegend). After 30 minutes of incubation in the dark, cells were washed, resuspended in Cell staining buffer, and measured on the FACSCanto II (BD Biosciences). Using the FlowJo V10 software (TreeStar Inc), we determined frequencies of IFN gamma–producing and IL-17A–producing T-cell subsets, expression of phospho-STATs in total CD4^+^ and CD8^+^ T cells from the whole blood, and the expression in the IFN gamma–positive and IL-17A–positive T-cell subsets. Our gating strategy is illustrated in Supplementary Figure 2 (see Supplementary Digital Content, http://links.lww.com/CTG/A950). All antibodies were titrated in preceding experiments. Fluorescence minus one and isotype controls were performed in all experiments.

### Analysis of phospho-STAT1 and phospho-STAT3 in biliary samples

Biliary tissue samples of patients with PSC were collected using a biopsy forceps during ERC and processed, as reported by von Seth et al (30). This allowed us to obtain more immune cells and more peribiliary tissue than with brush cytology. Tissue samples were analyzed by immunohistochemistry and multicolor flow cytometry.

### Phospho-STAT1/3 detection in bile duct tissue through immunohistochemistry

Immunostaining was performed on sections (2 μm) of formalin-fixed, paraffin-embedded ERC-derived tissue. All slides were processed on a BenchMark Ultra automated immunostainer (Ventana Medical Systems, Tucson, AZ) according to the manufacturer's instructions. The following primary antibodies were used: anti-phosphorylated STAT1 (ab30645, Abcam, Cambridge, UK) and anti-phosphorylated STAT3 (ab76315, Abcam). Positive reactions were visualized through oxidation of diaminobenzidine resulting in brown staining. Finally, sections were counterstained with hematoxylin, and image acquisition was performed with a Leica microscope. Immune cells and epithelial cells were identified by morphology. Nontumor-bearing tissue from 6 hepatic resections of hepatocellular carcinoma served as controls. All histopathology specimens were reviewed by 3 expert pathologists.

### Analysis of phospho-STAT1/3–positive cells through flow cytometry

Then, we used multicolor flow cytometry to identify the types of phospho-STAT–positive cells in bile duct specimens. In brief, the tissue was transferred immediately in a tube containing Roswell Park Memorial Institute 1640 medium (PAA Laboratories) and 10% FCS (ThermoFisher Scientific, Germany). Then, the tube was centrifuged, and the pellet was enzymatically digested with collagenase IV (Merck, Darmstadt, Germany), HS-Nuclease (MoBiTec, Berkheim, Germany), and broad bean trypsin inhibitor (PanBiotech, Aidenbach, Germany). After a 10-minute incubation on a shaker at room temperature, digested tissue and digestion medium were pressed through a cell strainer with a sterile rod, collected in a new tube and centrifuged again. Frequencies of phospho-STAT–positive cells in IFN gamma–positive and IL-17A–positive CD4^+^ T cells were analyzed as described earlier.

### *In vitro* inhibition of STAT3 phosphorylation and cytokine production in immune cells from patients with PSC by JAK inhibitors

Finally, we studied inhibitory activities of JAK inhibitors baricitinib, upadacitinib, and fedratinib in patients with PSC using the protocol of Kitanaga et al (31). To this end, we first analyzed frequencies of phospho-STAT3^+^ cells in the IFN gamma–positive and IL-17A–positive CD4^+^ T-cell subsets after stimulation of PBMCs with recombinant IL-6 when varied concentrations of JAK inhibitors (0–1,000 nM) had been added before. Next, we also studied the inhibition of IFN gamma, IL-17A, and IL-6 production in the supernatant of anti-CD3/anti-CD28–stimulated PBMCs using the LEGENDplex HU Essential Immune Response Panel.

### Statistical analysis

Statistical analysis was performed with SPSS (version 24; IBM Deutschland GmbH, Ehningen, Germany) and GraphPad Prism (version 8.0; GraphPad Prism, San Diego, CA). Datasets were first tested for normality and then analyzed with the Mann-Whitney *U* test, Wilcoxon matched-pairs signed rank test, and Student *t* test, as appropriate. Correlations between cytokine levels and activation of STATs in immune cells and correlations between experimental results and clinical data were compared by Spearman correlation coefficients. *In vitro* inhibition experiments were analyzed by a four-parameter logistic regression model using nonlinear least-squares curve fitting, from which IC50 values were estimated.

## RESULTS

### Frequencies of IFN gamma–positive and IL-17A–positive CD4^+^ T cells are increased in the blood and biliary tissue from patients with PSC

**Figure 1.**
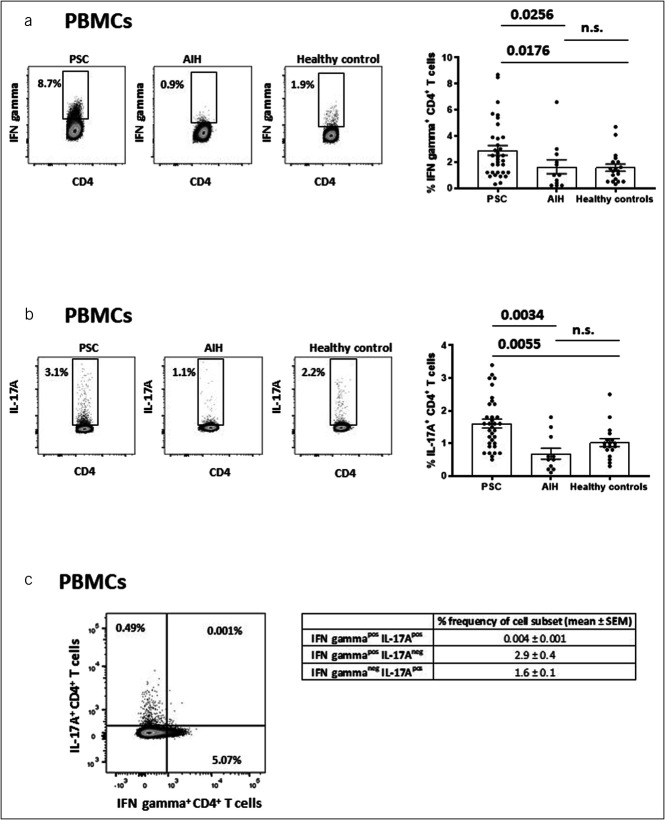
Frequencies of IFN gamma–positive and IL-17A–positive CD4^+^ T cells in PBMCs. These figures demonstrate that frequencies of IFN gamma-positive (**a**) and IL-17A-positive CD4^+^ T cells (**b**) in unstimulated PBMCs were higher in patients with PSC (n = 34) compared with patients with AIH (n = 12) and healthy controls (n = 19). (**a**) and (**b**) illustrate representative flow cytometric dot plots (left side) and summary statistics in each cohort (right side). (**c**) illustrate that IFN gamma–producing and IL-17A–producing CD4^+^ T cells represented 2 separate T-cell subpopulations rather than a single double-positive CD4^+^ T-cell population in PSC (n = 34). Boxes and whiskers indicate mean and SEM, respectively. *P* values were calculated pairwise by the nonparametric Wilcoxon test. AIH, autoimmune hepatitis; IL, interleukin; IFN, interferon; PBMC, peripheral blood mononuclear cell; PSC, primary sclerosing cholangitis.

In a first step, we analyzed frequencies of IFN gamma–producing and IL-17A–producing CD3^+^ T-cell subsets in unstimulated PBMCs of patients with PSC and compared them with patients with AIH and healthy controls. We found greater frequencies of IFN gamma–positive (Figure 1a) and IL-17A–positive CD4^+^ T cells (Figure 1b) in PBMCs from patients with PSC than in PBMCs from patients with AIH and healthy blood donors, respectively. Such differences were not found in CD8^+^ T-cell subsets (see Supplementary Figures 3A and 3B, Supplementary Digital Content, http://links.lww.com/CTG/A950).

In various inflammatory and autoimmune disorders, a novel type of IFN gamma/IL-17A–double-positive T cells has recently been described (32). However, in PSC, IFN gamma–producing and IL-17A–producing T cells represented 2 separate T-cell subpopulations (Figure 1c).

**Figure 2.**
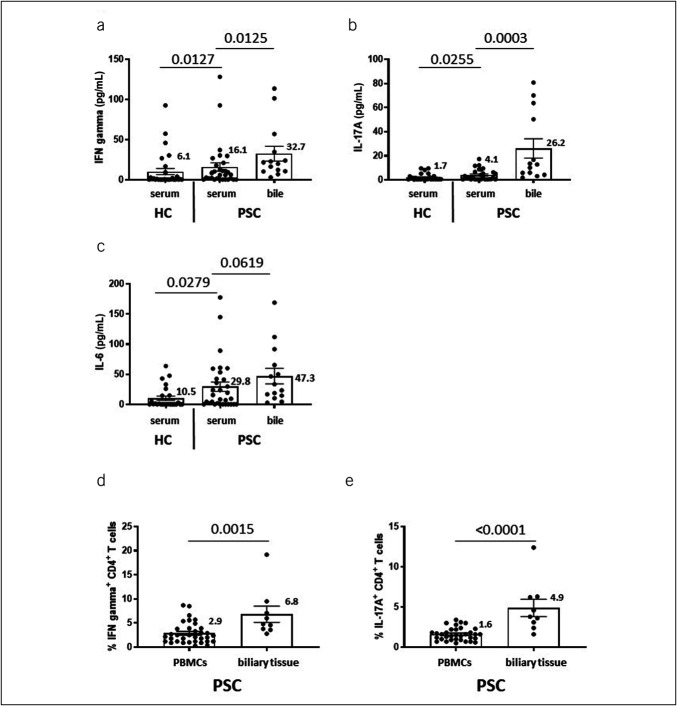
Levels of proinflammatory cytokines and frequencies of IFN gamma–positive and IL-17A–positive CD4^+^ T cells in PBMCs and biliary tissue. These figures compared cytokine levels between the serum (n = 30) and bile (n = 14) (**a** and **b**) and frequencies of proinflammatory CD4^+^ T-cell subsets between PBMCs (n = 34) and biliary tissue samples (n = 9) (**c** and **d**) without further stimulation. In PSC, levels of IFN gamma (**a**), IL-17A (**b**), and IL-6 in bile (**c**) exceeded those in the serum. Correspondingly, in the biliary tissue, frequencies of IFN gamma–producing (**d**) and IL-17A–producing CD4^+^ T cells (**e**) were higher than in PBMCs. Healthy controls in figures A, B, and C are shown to provide a frame of reference. Boxes and whiskers indicate mean and SEM, respectively. Numbers indicate mean values. *P* values were calculated pairwise by the nonparametric Wilcoxon test. HC, healthy control; IFN, interferon; IL, interleukin; PBMC, peripheral blood mononuclear cell; PSC, primary sclerosing cholangitis; SEM, standard error of the mean.

Because our key findings concerned CD4^+^ T cells in PSC, we focused further studies on patients with PSC. Corresponding to their increased frequencies of cytokine-producing CD4^+^ T cells in PBMCs (Figures 1a,b), we first confirmed increased serum levels of IFN gamma (Figure 2a), IL-17A (Figure 2b), and IL-6 (Figure 2c) in patients with PSC compared with those in healthy controls. Further analysis revealed that levels of IFN gamma (Figure 2a), IL-17A in bile (Figure 2b), and IL-6 (Figure 2c) exceeded those in the serum from patients with PSC. Correspondingly, in patients with PSC, frequencies of IFN gamma–producing (Figure 2d) and IL-17A–producing CD4^+^ T cells in biliary tissue (Figure 2e) were 2-fold higher than in PBMCs.

### STAT3 activation in PSC is upregulated in the whole blood and biliary tissue

**Figure 3.**
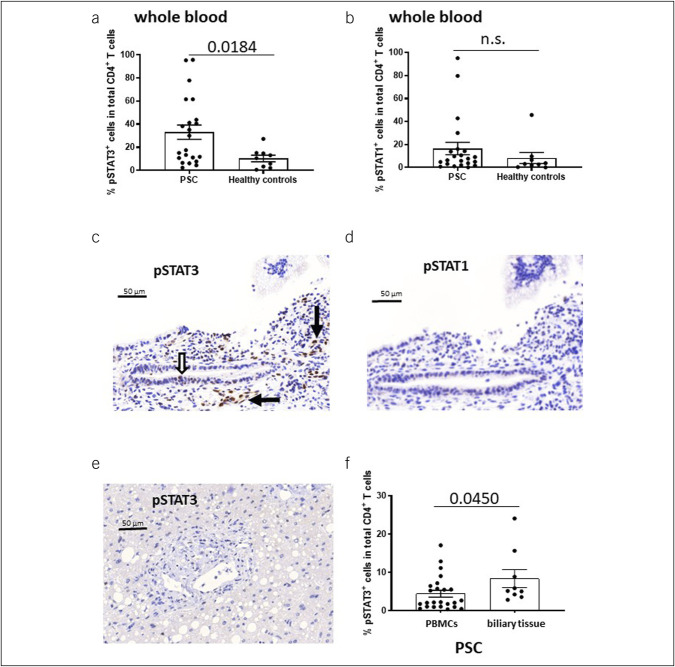
STAT3 activation in the whole blood and biliary tissue. (**a**) illustrates that frequencies of phospho-STAT3–positive cells in total CD4^+^ T cells of the whole blood after red blood cell lysis (n = 22) are higher in those with PSC than in healthy controls (n = 9). Such differences were not seen for phospho-STAT1 expression in the CD4^+^ T cells (PSC [n = 22] vs healthy controls [n = 9]) (**b**). (**c**) and (**d**) show immunohistochemical *in situ* analysis (100×) of phospho-STAT3 and phospho-STAT1 expression in the liver of patients with PSC (n = 5). Representative high-power fields illustrate expression of phospho-STAT3 in the portal stroma (black arrows in **c**) and moderate expression in cells of the biliary epithelium (white arrows in **c**). By contrast, expression of phospho-STAT1 in the stroma and biliary epithelium was only faint (**d**). (**e**) shows a representative phospho-STAT3 immunohistochemical staining of a portal field in the nontumor-bearing tissue of a control patient with hepatic steatosis and hepatocellular carcinoma. Of note, unlike PSC, phospho-STAT3 expression was not detected in any of the non-PSC control samples (n = 6). (**f**) demonstrates that in the biliary tissue, frequencies of phospho-STAT3–positive cells in total CD4^+^ T cells exceeded those in PBMCs. Boxes and whiskers indicate mean and SEM, respectively. Numbers indicate mean values. *P* values were calculated pairwise by the nonparametric Wilcoxon test. IFN, interferon; PBMC, peripheral blood mononuclear cell; PSC, primary sclerosing cholangitis.

To correlate the production of inflammatory cytokines to differential phosphorylation of STAT proteins, we first analyzed the expression of phospho-STAT1 and phospho-STAT3 in total CD4^+^ and CD8^+^ T cells in whole blood samples after RBC lysis. In PSC, we observed higher frequencies of phospho-STAT3 expression in total CD4^+^ T cells than in healthy controls (Figure 3a). By contrast, such differences were not seen for phospho-STAT1 expression in CD4^+^ T cells (Figure 3b) nor for expression of either phospho-STATs in CD8^+^ T cells (see Supplementary Figures 4A and 4B, Supplementary Digital Content, http://links.lww.com/CTG/A950).

Having identified increased frequencies of phospho-STAT3 expression in CD4^+^ T cells as a feature characteristic for PSC, we studied phospho-STAT3 expression in biliary biopsies. First, we studied *in situ* localization of phospho-STAT–positive cells in the bile duct tissue of patients with PSC. Our immunohistochemical studies revealed strong phospho-STAT3 immunoreactivity in the stroma (median 29 cell/high-power field [HPF], range: 5–51), but moderate expression of cells in the bile ducts (median 6 cells/HPF, range: 2–14) (Figure 3c). Of note, only faint expression of phospho-STAT1 was observed in the stroma (median 2 cells/HPF, range: 1–9) and biliary epithelium (median 5 cells/HPF, range: 1–23) (Figure 3d). Unlike PSC, immunohistochemistry did not reveal phospho-STAT3 expression in the nontumor-bearing tissue obtained from hepatic resections of hepatocellular carcinoma representing apparently normal liver histology or hepatic steatosis only (Figure 3e).

**Figure 4.**
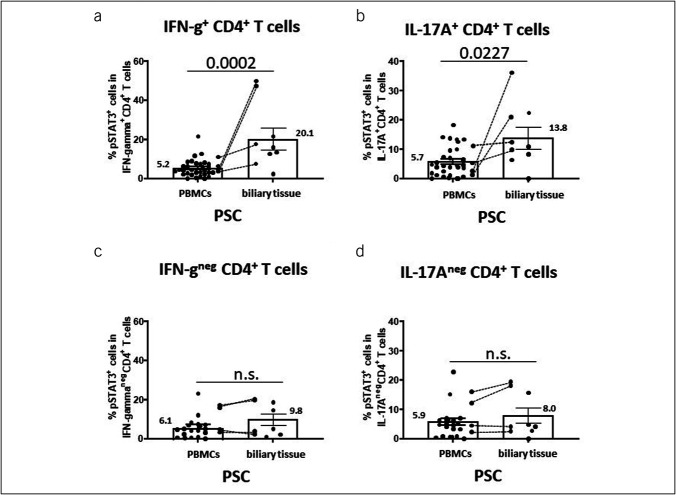
Increased frequencies of phospho-STAT3 expression in cytokine-producing CD4^+^ T-cell subsets in PSC. Figures illustrate that phospho-STAT3–positive CD4^+^ T cells with prominent production of interferon-gamma (**a**) and IL-17A (**b**) are particularly enriched in the peribiliary liver tissue (n = 9) compared with PBMCs in PSC (n = 25). By contrast, such differences were not seen for cytokine-negative CD4^+^ T-cell subsets (**c** and **d**). Paired samples are connected by dotted lines. Boxes and whiskers indicate mean and SEM, respectively. Numbers indicate mean values. *P* values were calculated pairwise by the nonparametric Wilcoxon test. IL, interleukin; PBMC, peripheral blood mononuclear cell; PSC, primary sclerosing cholangitis.

Next, we extracted vital cells from the biliary tissue and identified the type of phospho-STAT3–positive cells by flow cytometry. This analysis further confirmed that in biliary tissue frequencies of phospho-STAT3–positive cells in total CD4^+^ T cells exceeded those in PBMCs (Figure 3f). Of note, additional analysis of phospho-STAT3 expression in CD4^+^ T-cell subsets with cytokine production versus without cytokine production demonstrated that phospho-STAT3–positive CD4^+^ T cells with prominent production of IFN gamma (Figure 4a) and IL-17A (Figure 4b) are particularly enriched in the peribiliary liver tissue in PSC compared with PBMCs. By contrast, such preferential localization was not seen for cytokine-negative CD4^+^ T cells (Figure 4c,d).

#### In PSC, IL-6 enhances phospho-STAT3 expression in inflammatory CD4^+^ T cells

**Figure 5.**
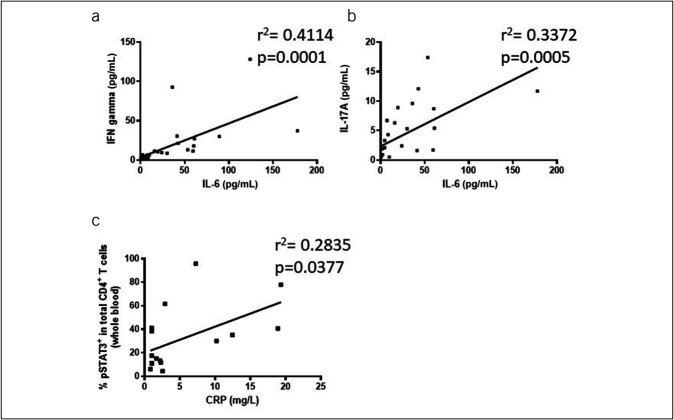
IL-6, IFN gamma, and IL-17A in the serum and the relationship to phospho-STAT3^+^ CD4^+^ T cells and CRP in PSC. In PSC, IL-6 levels were correlated with serum levels of both IFN gamma (n = 31) (**a**) and IL-17A (n = 31) (**b**) indicating interactions between the various proinflammatory cytokines. (**c**) illustrates that frequencies of phospho-STAT3–positive total CD4^+^ T cells in the whole blood correlate to serum CRP levels (n = 15). *P* values were calculated by the nonparametric Wilcoxon test, and correlation coefficients refer to Spearman rank correlation coefficients. CRP, C-reactive protein; IL, interleukin; IFN, interferon.

Because activation of STAT1 and STAT3 lead to the induction of proinflammatory responses, we studied putative relations between the various cytokines. Unlike healthy controls, IL-6 serum levels in PSC were correlated with serum levels of both IFN gamma (Figure 5a) and IL-17A (Figure 5b) indicating interactions between the various proinflammatory cytokines. Finally, frequencies of total phospho-STAT3–positive CD4^+^ T cells in the whole blood after RBC lysis correlated to IL-6 serum levels (r^2^ = 0.6803, *P* = 0.0433) and more importantly also serum C-reactive protein levels (Figure 5c).

**Figure 6.**
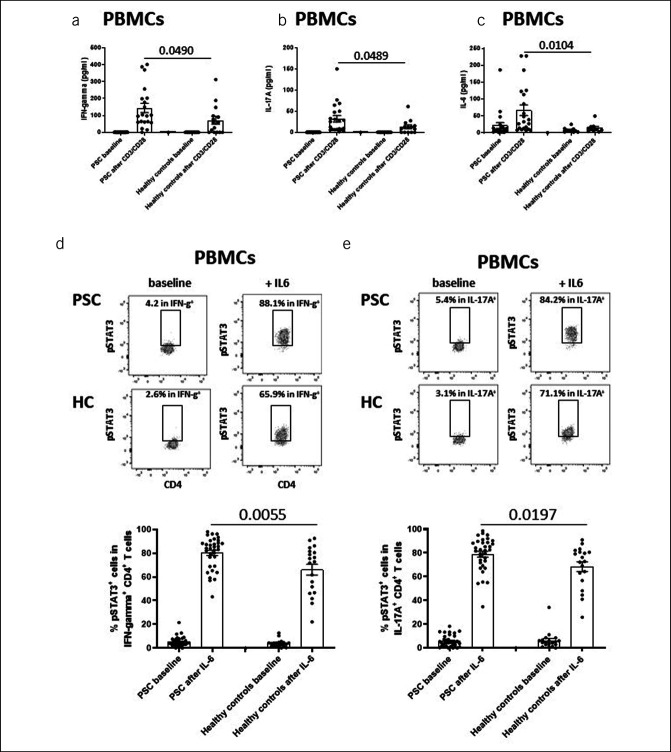
Cytokine levels and frequencies of phospho-STAT3–positive cells in IFN gamma^+^ and IL-17A^+^ CD4^+^ T cells after *in vitro* stimulation of PBMCs. (**a**–**c**) demonstrate that maximal stimulation of T-cell receptors in PBMCs with anti-CD3 (1 μg/mL) plus anti-CD28 (0.5 μg/mL) resulted in greater amounts of IFN gamma, IL-17A, and IL-6 in PBMCs from patients with PSC (n = 20) compared with healthy controls (n = 15). Likewise, *in vitro* stimulation with recombinant IL-6 (50 ng/mL) resulted in greater numbers of phospho-STAT3–positive cells in the IFN gamma–positive (**d**) and IL-17A–positive CD4^+^ T-cell subsets (**e**) in patients with PSC (n = 34) than in healthy controls (n = 19). Upper parts of (**d**) and (**e**) illustrate representative dot plots of phospho-STAT3–positive cells in IFN gamma–producing and IL-17A–producing CD4^+^ T cells in patient with PSC #6 and healthy control #1 at baseline and after IL-6 stimulation. The lower parts of (**d**) and (**e**) summarize results of all patients with PSC and healthy controls. Boxes and whiskers indicate mean values and SEM, respectively. *P* values were calculated by the nonparametric Wilcoxon test. HC, healthy controls; IFN, interferon; IL, interleukin; PBMC, peripheral blood mononuclear cell; PSC, primary sclerosing cholangitis.

Next, we studied cytokine release on a broad *in* vitro stimulation of PBMCs with anti-CD3/CD28, which mimics T-cell receptor stimulation and enables activation of several signaling pathways rather than IL-6/STAT3 alone. In this study, T-cell receptor stimulation resulted in greater amounts of IFN gamma (Figure 6a), IL-17A (Figure 6b), and IL-6 (Figure 6c) in PBMCs from patients with PSC compared with those from healthy controls. Of importance, *in vitro* stimulation of PBMCs with IL-6 resulted in greater numbers of phospho-STAT3–positive cells in the IFN gamma-positive (Figure 6d) and IL-17A–positive CD4^+^ T-cell subsets (Figure 6e) of patients with PSC compared with those in healthy controls. Of note, IL-6 potently activates STAT1 in CD4^+^ T cells and STAT1/3 in CD8^+^ T cells, but there was no significant difference between patients with PSC and healthy controls (see Supplementary Figures 5A–5F, Supplementary Digital Content, http://links.lww.com/CTG/A950). These findings suggest that in PSC, IL-6 stimulates enhanced STAT3 activation and production of proinflammatory cytokines in CD4^+^ T cells.

We addressed several potential confounding factors: First, we compared patients with PSC with IBD with those without IBD but could not find differences between the 2 patient groups concerning serum levels of cytokines, frequencies of proinflammatory CD4^+^ T cells in unstimulated PBMCs, and numbers of phospho-STAT3^+^ IFN gamma–producing and IL-17A–producing CD4^+^ T cells after *in vitro* stimulation of PBMCs with IL-6 (see Supplementary Figures 6A–6F, Supplementary Digital Content, http://links.lww.com/CTG/A950). Furthermore, we stratified and reanalyzed our patients regarding medication. However, neither mesalazine (see Supplementary Figure 7A, Supplementary Digital Content, http://links.lww.com/CTG/A950) nor UDCA (see Supplementary Figure 7B, Supplementary Digital Content, http://links.lww.com/CTG/A950) affected our results.

### Inhibition of *in vitro* cytokine induction by JAK inhibitors

**Figure 7.**
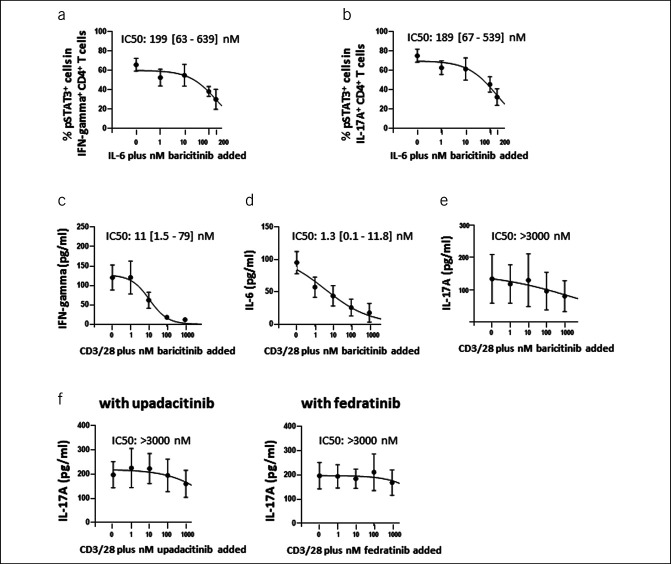
Inhibition of *in vitro* cytokine induction by JAK inhibitors. These figures demonstrate that baricitinib (JAK1/2 inhibitor) blocked IL-6–induced expression of phospho-STAT3 in interferon gamma^+^ (**a**) and IL-17A^+^ CD4^+^ cells in PBMCs from patients with PSC (**b**) with equal efficacy. In addition, baricitinib inhibition corresponded to reduced levels of IFN gamma (**c**) and IL-6 production (**d**) in anti-CD3/anti-CD28–stimulated PBMCs, while production of IL-17A was more resistant (**e**). Poor inhibition of IL-17A to JAK1/JAK2 inhibitors is confirmed when upadacitinib (selective blocker of JAK1; **f**) and fedratinib (selective blocker of JAK2; **g**) were studied, respectively. Inhibition experiments (n > 3) were analyzed by a 4-parameter logistic regression model using nonlinear least-squares curve fitting to estimate IC50 values. IL, interleukin; JAK, janus kinase; PBMC, peripheral blood mononuclear cell; PSC, primary sclerosing cholangitis.

Mutual complex regulatory circuits of STAT activation lead to amplified inflammation (17,21,33,34). To clarify this issue, we performed *in vitro* inhibition experiments with PBMCs and baricitinib, a selective JAK1 and JAK2 inhibitor with moderate activity against TYK2 (35,36). These studies demonstrated that baricitinib blocked IL-6–induced expression of phospho-STAT3 in IFN gamma^+^ (IC50:199 nM; Figure 7a) and IL-17A^+^ CD4^+^ cells with equal efficacy (IC50: 189 nM; Figure 7b). Of note, baricitinib inhibition corresponded to reduced levels of IFN gamma (IC50: 11 nM; Figure 7c) and IL-6 production (IC50: 1.3 nM; Figure 7d) in T-cell receptor–stimulated immune cells, while production of IL-17A was more resistant (IC50: >3,000 nM; Figure 7e). Poor inhibition of IL-17A to JAK1/JAK2 inhibitors was confirmed when upadacitinib (selectively blocks JAK1; Figure 7f left) and fedratinib (selectively blocks JAK2; Figure 7f right) were studied, respectively.

## DISCUSSION

Periductal inflammation and fibrosis is the histopathological hallmark of PSC, and previous studies have proposed proinflammatory TH1 (6,7) and TH17 cells as pivotal underlying inflammatory mediators (8,9). However, details of the inflammatory interactions in PSC are still poorly understood. In line with previous reports, our findings largely support the concept of IL-6–dependent STAT3 activation of proinflammatory CD4^+^ T cells in PSC.

We found increased frequencies of IFN gamma-positive and IL-17A-positive CD4^+^ T cells in PBMCs and high concentrations of IFN gamma, IL-17A, and IL-6 in the serum of patients with PSC. Frequencies of IL-17A–positive CD4^+^ T cells corresponded to previous reports, but numbers of IFN gamma–positive CD4^+^ T cells tended to be a bit lower than previously reported (6–8,13). This difference is explained by the fact that we studied cell frequencies without stimulation, an experimental approach, which in various infections has already been shown to reflect ongoing *in situ* activation (37,38).

Of interest, dual IL-17A–positive and IFN gamma–positive T cells have been reported in various fibrosing autoimmune diseases (32). In systemic scleroderma, such IFN gamma/IL-17A double-positive cells could be linked directly to fibrosis regulation through IL-21 release (39), and in PSC, expansion of IFN gamma coexpressing TH17 cells was observed after *in vitro* stimulation with bacterial antigens (8). However, in our patients with PSC, CD4^+^ T cells represented distinctive subsets, which either produced IFN gamma or IL-17A. In line with our observations, double-positive T cells represented only a minor fraction in the CD161^+^ CD4^+^ T-memory cell subset in patients with IBD (40,41). This suggests that TH17 cells do not present a “fixed subset” but show considerable plasticity leading to various subsets depending on the underlying disease and the microenvironment (32,42).

Of importance, our flow cytometry analysis further demonstrated that in PSC, proinflammatory CD4^+^ T cells and their cytokines IFN gamma, IL-17A, and IL-6 were more pronounced in the bile and peribiliary liver tissue when compared with the blood suggesting local production and a pivotal role for the local inflammatory immune response around bile ducts. These observations are supported by several reports on liver specimens from non-PSC controls, which consistently reported that cytokines IFN gamma, IL-17A, and IL-6 were exclusively detectable when cholangitis was present (43–45).

Inflammation in PSC was related to phospho-STAT3 activation so that expanded phospho-STAT3–positive CD4^+^ T-cell subsets with IFN gamma and IL-17A production were a key finding in the blood and the liver. By contrast, phosphorylation of STAT1 was apparently not enhanced probably also reflecting a mutual inverse balance between STAT1 and STAT3 activation as previously reported (46). In particular, local enrichment of phospho-STAT3–positive inflammatory cells in the biliary tissue is supported by our flow cytometric analysis of biliary biopsies demonstrating that phospho-STAT3–positive cells were enriched in these tissue samples while histology showed STAT3-positive cells in the stroma of portal tracts around the bile ducts, which is in line with previous histological reports on the distribution of IFN gamma–positive and IL-17A–positive cells (13).

Network-based search for molecular circuits identified prominent *STAT3* gene expression in patients with cholangiopathies including PSC (12) and in those developing CCA (47). In line with such studies, we found elevated IL-6 serum levels, which corresponded to IFN gamma and IL-17A and frequencies of phospho-STAT3–expressing cells in total CD4^+^ T cells. Beyond that, we found that in PSC, IL-6 stimulation resulted in increased frequencies of phospho-STAT3^+^ cells in the IFN gamma–positive and IL-17A–positive CD4^+^ T-cell subsets suggesting presumably enhanced IL-6 susceptibility of inflammatory CD4^+^ T cells.

It has been claimed that inflammatory T cells in PSC might be triggered by microbial components in patients with a somewhat “leaky” gut and become homed to the stroma around bile ducts thereafter (48). In particular, segmented filamentous bacteria can adhere to intestinal epithelial cells and induce TH17 cells in murine and human gut (49,50). However, in line with other investigators, we did not find differences between patients with PSC with and without concomitant IBD confirming that induction of proinflammatory T cells occurs independently from the presence of IBD (8,14). On the contrary, we found a correlation of phospho-STAT3^+^ CD4^+^ T cells to serum C-reactive protein, a surrogate marker of systemic inflammation, which has been linked to disease prognosis in a variety of cardiovascular, metabolic, autoimmune, and malignant diseases. Thus, JAK-STAT3 activation and systemic inflammation are likely to affect prognosis also in PSC.

To date, no medical therapy has proven unequivocal benefit concerning clinical outcomes of PSC (5), but dysregulation in the JAK-STAT pathway is considered an important step in the pathogenesis of several inflammatory and autoimmune diseases with a prominent IL-6, IFN gamma, and IL-17A signature (20). Selective JAK inhibitors may offer promising novel treatment options and are currently evaluated for the treatment of several immune-mediated diseases such as rheumatoid arthritis (51) and IBD (52). To address the potential impact of such drugs in PSC, we studied *in vitro* JAK1/2 inhibition of immune cells from patients with PSC using the broadly active inhibitor baricitinib and selective JAK1 and JAK2 inhibitors upadacitinib and fedratinib, respectively. We observed the inhibition of STAT3 phosphorylation and IL-6 and IFN gamma secretion by CD4^+^ T cells on T-cell receptor stimulation with anti-CD3/CD28 in the expected low nM range (53). By contrast, however, IL-17A turned out to be rather refractory to inhibition of JAK1/2. These findings indicate that immune regulation in PSC is more complex. In particular, activation of IL-17A is likely to also involve other alternative signaling pathways beyond JAK1/2-STAT3 such as IL-23 or other factors (42,54). Identification of these additionally involved pathways in future studies may allow for better therapeutic targeting of PSC.

Of interest, we did not observe enhanced activation of IFN gamma and IL-17A–producing CD4^+^ T cells in AIH. However, all our patients with AIH received immunosuppressive therapy. Thus, we cannot exclude that STAT3 phosphorylation and IFN gamma and IL-17A production is activated in patients with untreated AIH. However, our data show that current therapies do not prevent inflammatory activity in PSC, and from our *in vitro* experiments, we conclude that even pharmacological JAK-STAT3 blockade cannot fully control inflammation in the presence of broad T-cell activation such as anti-CD3/CD28 stimulation. Because reliable surrogate markers to assess PSC progression have not yet been identified, interpretation of immunological data concerning the clinical disease course remains difficult (55). Unfortunately, we could not directly study the relationship between STAT3 phosphorylation and PSC disease progression because clinical events such as death, transplantation, CCA, and recurrent cholangitis were infrequent in our patients. However, we found that the presence of IBD was not associated with differential STAT3 phosphorylation, and activation of the STAT3 pathway in PSC was not altered by UDCA and mesalazine treatment.

Overall, our data support the concept of increased IL-6–dependent activation of transcription factor STAT3 in PSC, which leads to enhanced production of IFN gamma and IL-17A in CD4^+^ T cells and increased systemic inflammation. This process seems to be particularly prominent in the peribiliary liver tissue. Thus, our results suggest local and systemic activation of the IL-6/STAT3 pathway in PSC. However, *in vitro* blocking experiments demonstrated that JAK-STAT inhibition alone is not sufficient to completely control inflammatory activity in PSC. Thus, a search for further immune activation pathways is warranted in PSC.

## CONFLICTS OF INTEREST

**Guarantor of the article:** Bettina Langhans, Dr.rer.nat.

**Specific author contributions:** L.D., U.S., and B.L.: study concept and design. L.D., L.F., P.L., D.J.K., T.W., V.B., M.G.-C., C.P.S., B.L.: acquisition of patients and healthy controls. L.D., M.T.: immunohistochemistry analysis. L.D., L.F., B.K., J.N., V.B., M.T., U.S., B.L.: collection, analysis, and interpretation of the data. L.D., U.S., B.L.: drafting of the manuscript. All authors have read and approved the final version of this manuscript.

**Financial support:** L.F. and B.L.: received a grant from the EKFS-Promotionskolleg „NeuroImmunology“, Bonn. Grant number 2020-S2-3. This work was supported by the Open Access Publication Fund of the University of Bonn.

**Potential competing interests:** None to report.

**Study Highlights**

**WHAT IS KNOWN**

✓ Primary sclerosing cholangitis (PSC) is a rare cholestatic liver disease with periductal inflammation and fibrosis.
✓ Proinflammatory T cells play an essential role in PSC pathogenesis.
✓ IL-6–dependent immune cell activation has been suggested by genetic studies.

**WHAT IS NEW HERE**

✓ IL-6 triggers the phosphorylation of STAT3 in T cells leading to enhanced production of IFN gamma and IL-17A in PSC.
✓ Phospho-STAT3–positive T cells correlate with systemic inflammation and are particularly enriched in peribiliary liver tissue.
✓ Local and systemic activation of the IL-6/STAT3 pathway contributes to PSC pathogenesis. However, further pathways must be involved to explain IL-17A induction, which persisted despite inhibition of phospho-STAT3 activation.

## References

[1] Boonstra K Beuers U Ponsioen CY. Epidemiology of primary sclerosing cholangitis and primary biliary cirrhosis: A systematic review. J Hepatol. 2012;56:1181–8. 22245904 10.1016/j.jhep.2011.10.025

[2] Practice guideline autoimmune liver diseases—AWMF-Reg. No. 021-27 [in German]. Z Gastroenterol. 2017;55:1135–226. 29141269 10.1055/s-0043-120199

[3] Boberg KM Bergquist A Mitchell S Cholangiocarcinoma in primary sclerosing cholangitis: Risk factors and clinical presentation. Scand J Gastroenterol. 2002;37:1205–11. 12408527 10.1080/003655202760373434

[4] Tsaitas C Semertzidou A Sinakos E. Update on inflammatory bowel disease in patients with primary sclerosing cholangitis. World J Hepatol. 2014;6:178–87. 24799986 10.4254/wjh.v6.i4.178 PMC4009473

[5] Vesterhus M Karlsen TH. Emerging therapies in primary sclerosing cholangitis: Pathophysiological basis and clinical opportunities. J Gastroenterol. 2020;55:588–614. 32222826 10.1007/s00535-020-01681-z PMC7242240

[6] Liaskou E Jeffery LE Trivedi PJ Loss of CD28 expression by liver-infiltrating T cells contributes to pathogenesis of primary sclerosing cholangitis. Gastroenterology. 2014;147:221–32.e7. 24726754 10.1053/j.gastro.2014.04.003 PMC4961260

[7] Gwela A Siddhanathi P Chapman RW Th1 and innate lymphoid cells accumulate in primary sclerosing cholangitis-associated inflammatory bowel disease. J Crohns Colitis. 2017;11:1124–34. 28383652 10.1093/ecco-jcc/jjx050 PMC5637950

[8] Katt J Schwinge D Schoknecht T Increased T helper type 17 response to pathogen stimulation in patients with primary sclerosing cholangitis. Hepatology. 2013;58:1084–93. 23564624 10.1002/hep.26447

[9] Zimmer CL von Seth E Buggert M A biliary immune landscape map of primary sclerosing cholangitis reveals a dominant network of neutrophils and tissue-resident T cells. Sci Transl Med. 2021;13:eabb3107. 34162753 10.1126/scitranslmed.abb3107

[10] Xu B Broome U Ericzon BG High frequency of autoantibodies in patients with primary sclerosing cholangitis that bind biliary epithelial cells and induce expression of CD44 and production of interleukin 6. Gut. 2002;51:120–7. 12077104 10.1136/gut.51.1.120 PMC1773278

[11] Landi A Weismuller TJ Lankisch TO Differential serum levels of eosinophilic eotaxins in primary sclerosing cholangitis, primary biliary cirrhosis, and autoimmune hepatitis. J Interferon Cytokine Res. 2014;34:204–14. 24168449 10.1089/jir.2013.0075 PMC3942695

[12] Luo Z Jegga AG Bezerra JA. Gene-disease associations identify a connectome with shared molecular pathways in human cholangiopathies. Hepatology. 2018;67:676–89. 28865156 10.1002/hep.29504 PMC5834359

[13] Kunzmann LK Schoknecht T Poch T Monocytes as potential mediators of pathogen-induced T-helper 17 differentiation in patients with primary sclerosing cholangitis (PSC). Hepatology. 2020;72:1310–26. 33090557 10.1002/hep.31140

[14] Poch T Krause J Casar C Single-cell atlas of hepatic T cells reveals expansion of liver-resident naive-like CD4(+) T cells in primary sclerosing cholangitis. J Hepatol. 2021;75:414–23. 33774059 10.1016/j.jhep.2021.03.016 PMC8310924

[15] Aaronson DS Horvath CM. A road map for those who don't know JAK-STAT. Science. 2002;296:1653–5. 12040185 10.1126/science.1071545

[16] Rawlings JS Rosler KM Harrison DA. The JAK/STAT signaling pathway. J Cell Sci. 2004;117:1281–3. 15020666 10.1242/jcs.00963

[17] Stark GR. How cells respond to interferons revisited: From early history to current complexity. Cytokine Growth Factor Rev. 2007;18:419–23. 17683974 10.1016/j.cytogfr.2007.06.013 PMC2081984

[18] Horvath CM. The Jak-STAT pathway stimulated by interferon gamma. Sci STKE. 2004;2004:tr8. 15561980 10.1126/stke.2602004tr8

[19] Durant L Watford WT Ramos HL Diverse targets of the transcription factor STAT3 contribute to T cell pathogenicity and homeostasis. Immunity. 2010;32:605–15. 20493732 10.1016/j.immuni.2010.05.003 PMC3148263

[20] Camporeale A Poli V. IL-6, IL-17 and STAT3: A holy trinity in auto-immunity?. Front Biosci (Landmark Ed). 2012;17:2306–26. 22652781 10.2741/4054

[21] Ogura H Murakami M Okuyama Y Interleukin-17 promotes autoimmunity by triggering a positive-feedback loop via interleukin-6 induction. Immunity. 2008;29:628–36. 18848474 10.1016/j.immuni.2008.07.018

[22] Isomaki P Junttila I Vidqvist KL The activity of JAK-STAT pathways in rheumatoid arthritis: Constitutive activation of STAT3 correlates with interleukin 6 levels. Rheumatology (Oxford). 2015;54:1103–13. 25406356 10.1093/rheumatology/keu430

[23] Atreya R Neurath MF. Involvement of IL-6 in the pathogenesis of inflammatory bowel disease and colon cancer. Clin Rev Allergy Immunol. 2005;28:187–96. 16129903 10.1385/CRIAI:28:3:187

[24] Yu H Pardoll D Jove R. STATs in cancer inflammation and immunity: A leading role for STAT3. Nat Rev Cancer. 2009;9:798–809. 19851315 10.1038/nrc2734 PMC4856025

[25] Murakami M Hirano T. A four-step model for the IL-6 amplifier, a regulator of chronic inflammations in tissue-specific MHC class II-associated autoimmune diseases. Front Immunol. 2011;2:22. 22566812 10.3389/fimmu.2011.00022 PMC3341963

[26] Halliday JS Djordjevic J Lust M A unique clinical phenotype of primary sclerosing cholangitis associated with Crohn's disease. J Crohns Colitis. 2012;6:174–81. 22325171 10.1016/j.crohns.2011.07.015

[27] Dignass A Eliakim R Magro F Second European evidence-based consensus on the diagnosis and management of ulcerative colitis part 1: Definitions and diagnosis. J Crohns Colitis. 2012;6:965–90. 23040452 10.1016/j.crohns.2012.09.003

[28] Van Assche G Dignass A Panes J The second European evidence-based consensus on the diagnosis and management of Crohn's disease: Definitions and diagnosis. J Crohns Colitis. 2010;4:7–27. 21122488 10.1016/j.crohns.2009.12.003

[29] Kemp TJ Castro FA Gao YT Application of multiplex arrays for cytokine and chemokine profiling of bile. Cytokine. 2015;73:84–90. 25743242 10.1016/j.cyto.2015.01.033 PMC4382212

[30] von Seth E Zimmer CL Reuterwall-Hansson M Primary sclerosing cholangitis leads to dysfunction and loss of MAIT cells. Eur J Immunol. 2018;48:1997–2004. 30252934 10.1002/eji.201847608

[31] Kitanaga Y Imamura E Nakahara Y In vitro pharmacological effects of peficitinib on lymphocyte activation: A potential treatment for systemic sclerosis with JAK inhibitors. Rheumatology (Oxford). 2020;59:1957–68. 31764973 10.1093/rheumatology/kez526 PMC7382595

[32] Kamali AN Noorbakhsh SM Hamedifar H A role for Th1-like Th17 cells in the pathogenesis of inflammatory and autoimmune disorders. Mol Immunol. 2019;105:107–15. 30502718 10.1016/j.molimm.2018.11.015

[33] Chen Z Laurence A Kanno Y Selective regulatory function of Socs3 in the formation of IL-17-secreting T cells. Proc Natl Acad Sci U S A. 2006;103:8137–42. 16698929 10.1073/pnas.0600666103 PMC1459629

[34] Wang L Yi T Kortylewski M IL-17 can promote tumor growth through an IL-6-Stat3 signaling pathway. J Exp Med. 2009;206:1457–64. 19564351 10.1084/jem.20090207 PMC2715087

[35] Fridman JS Scherle PA Collins R Selective inhibition of JAK1 and JAK2 is efficacious in rodent models of arthritis: Preclinical characterization of INCB028050. J Immunol. 2010;184:5298–307. 20363976 10.4049/jimmunol.0902819

[36] McInnes IB Byers NL Higgs RE Comparison of baricitinib, upadacitinib, and tofacitinib mediated regulation of cytokine signaling in human leukocyte subpopulations. Arthritis Res Ther. 2019;21:183. 31375130 10.1186/s13075-019-1964-1 PMC6679539

[37] Walker D Jason J Wallace K Spontaneous cytokine production and its effect on induced production. Clin Diagn Lab Immunol. 2002;9:1049–56. 12204958 10.1128/CDLI.9.5.1049-1056.2002 PMC120078

[38] Brunialti MK Santos MC Rigato O Increased percentages of T helper cells producing IL-17 and monocytes expressing markers of alternative activation in patients with sepsis. PLoS One. 2012;7:e37393. 22693573 10.1371/journal.pone.0037393 PMC3365066

[39] Xing X Li A Tan H IFN-gamma(+) IL-17(+) Th17 cells regulate fibrosis through secreting IL-21 in systemic scleroderma. J Cell Mol Med. 2020;24:13600–8. 33157566 10.1111/jcmm.15266 PMC7753990

[40] Kleinschek MA Boniface K Sadekova S Circulating and gut-resident human Th17 cells express CD161 and promote intestinal inflammation. J Exp Med. 2009;206:525–34. 19273624 10.1084/jem.20081712 PMC2699125

[41] Sakuraba A Sato T Kamada N Th1/Th17 immune response is induced by mesenteric lymph node dendritic cells in Crohn's disease. Gastroenterology. 2009;137:1736–45. 19632232 10.1053/j.gastro.2009.07.049

[42] Stadhouders R Lubberts E Hendriks RW. A cellular and molecular view of T helper 17 cell plasticity in autoimmunity. J Autoimmun. 2018;87:1–15. 29275836 10.1016/j.jaut.2017.12.007

[43] Zhou T Bartelheimer K Ruping F Intrahepatic biliary strictures after liver transplantation are morphologically similar to primary sclerosing cholangitis but immunologically distinct. Eur J Gastroenterol Hepatol. 2020;32:276–84. 31895887 10.1097/MEG.0000000000001649

[44] Rosen HR Winkle PJ Kendall BJ Biliary interleukin-6 and tumor necrosis factor-alpha in patients undergoing endoscopic retrograde cholangiopancreatography. Dig Dis Sci. 1997;42:1290–4. 9201097 10.1023/a:1018822628096

[45] Muller T Beutler C Pico AH Increased T-helper 2 cytokines in bile from patients with IgG4-related cholangitis disrupt the tight junction-associated biliary epithelial cell barrier. Gastroenterology. 2013;144:1116–28. 23391819 10.1053/j.gastro.2013.01.055

[46] Regis G Pensa S Boselli D Ups and downs: The STAT1:STAT3 seesaw of interferon and gp130 receptor signalling. Semin Cell Dev Bio.l 2008;19:351–9. 10.1016/j.semcdb.2008.06.004 18620071

[47] Dokduang H Techasen A Namwat N STATs profiling reveals predominantly-activated STAT3 in cholangiocarcinoma genesis and progression. J Hepatobiliary Pancreat Sci. 2014;21:767–76. 25044480 10.1002/jhbp.131

[48] Nakamoto N Sasaki N Aoki R Gut pathobionts underlie intestinal barrier dysfunction and liver T helper 17 cell immune response in primary sclerosing cholangitis. Nat Microbiol. 2019;4:492–503. 30643240 10.1038/s41564-018-0333-1

[49] Atarashi K Tanoue T Ando M Th17 cell induction by adhesion of microbes to intestinal epithelial cells. Cell. 2015;163:367–80. 26411289 10.1016/j.cell.2015.08.058 PMC4765954

[50] Chen B Ye D Luo L Adhesive bacteria in the terminal ileum of children correlates with increasing Th17 cell activation. Front Pharmacol. 2020;11:588560. 33390964 10.3389/fphar.2020.588560 PMC7774322

[51] Harrington R Al Nokhatha SA Conway R. JAK inhibitors in rheumatoid arthritis: An evidence-based review on the emerging clinical data. J Inflamm Res. 2020;13:519–31. 32982367 10.2147/JIR.S219586 PMC7500842

[52] Dudek P Fabisiak A Zatorski H Efficacy, safety and future perspectives of JAK inhibitors in the IBD treatment. J Clin Med. 2021:10. 34884361 10.3390/jcm10235660 PMC8658230

[53] Hu X Li J Fu M The JAK/STAT signaling pathway: From bench to clinic. Signal Transduct Target Ther. 2021;6:402. 34824210 10.1038/s41392-021-00791-1 PMC8617206

[54] Yasuda K Takeuchi Y Hirota K. The pathogenicity of Th17 cells in autoimmune diseases. Semin Immunopathol. 2019;41:283–97. 30891627 10.1007/s00281-019-00733-8

[55] Ponsioen CY Chapman RW Chazouilleres O Surrogate endpoints for clinical trials in primary sclerosing cholangitis: Review and results from an International PSC Study Group consensus process. Hepatology. 2016;63:1357–67. 26418478 10.1002/hep.28256

